# Prediction of imminent osteoporotic fracture risk in Danish postmenopausal women—can the addition of self-reported clinical risk factors improve the prediction of the register-based FREM algorithm?

**DOI:** 10.1007/s11657-024-01493-1

**Published:** 2025-02-07

**Authors:** Emilie Rosenfeldt Christensen, Kasper Westphal Leth, Frederik Lykke Petersen, Tanja Gram Petersen, Sören Möller, Bo Abrahamsen, Katrine Hass Rubin

**Affiliations:** 1 Research Unit OPEN, Department of Clinical Research, University of Southern Denmark, Odense, Denmark; 2 OPEN - Open Patient data Explorative Network, Odense University Hospital, Odense, Denmark; 3 Department of Medicine, Holbæk Hospital, Holbæk, Denmark

**Keywords:** Osteoporosis, Risk prediction, Fracture, Case finding, Prediction model

## Abstract

***Summary*:**

Obtaining accurate self-reports on clinical risk factors, such as parental hip fracture or alcohol and tobacco use, limits the utility of conventional risk scores for fracture risk. We demonstrate that fracture-risk prediction based on administrative health data alone performs equally to prediction based on self-reported clinical risk factors.

**Background:**

Accurate assessment of fracture risk is crucial. Unlike established risk prediction tools that rely on patient recall, the Fracture Risk Evaluation Model (FREM) utilises register data to estimate the risk of major osteoporotic fracture (MOF). We investigated whether adding self-reported clinical risk factors for osteoporosis to the FREM algorithm improved the prediction of 1-year fracture risk by comparing three approaches: the FREM algorithm (FREM^orig^), clinical risk factors (CRF^only^), and FREM combined with clinical risk factors (FREM-CRF).

**Method:**

Clinical risk factor information was obtained through questionnaires sent to women aged 65–80 years living in the Region of Southern Denmark in 2010, who participated in the Risk-stratified Osteoporosis Strategy Evaluation study. Register data was obtained through national health registers and linked to the survey data. Positive and negative predictive values and concordance statistics were calculated for the performance of each approach using logistic regression and Cox proportional hazards models.

**Results:**

Of the 18,605 women included, 280 sustained a MOF within 1 year. All three approaches performed similarly in 1-year fracture risk prediction for low- and high-risk individuals. However, the FREM^orig^ and FREM-CRF approach slightly overestimated fracture risk for medium-risk individuals.

**Conclusion:**

Adding self-reported clinical data to FREM did not increase precision in predicting 1-year MOF risk. The discrimination of FREM^orig^ was similar to that of CRF^only^, suggesting it may be possible to estimate fracture risk with the same precision by using register data instead of self-reported risk information. Register-based prediction models may be applicable in individualised risk monitoring or large-scale osteoporosis screening programmes.

**Supplementary Information:**

The online version contains supplementary material available at 10.1007/s11657-024-01493-1.

## Background

Osteoporosis and osteoporotic fractures represent a growing public health challenge. The fractures affect a large portion of the elderly and place a significant economic burden on society, in addition to the debilitating consequences faced by individuals [1]. Loss of independence, poorer quality of life, increased morbidity, and death all occur as a result of osteoporotic fractures, and the number of new fractures is expected to rise as the elderly population grows [2–5]. Despite guidelines on the diagnosis and management of osteoporosis and the availability of effective anti-osteoporotic medication, the disease remains both underdiagnosed and undertreated [5–8].

Accurate assessment of fracture risk is crucial in targeted interventions to prevent fractures. As reviewed elsewhere, risk estimation tools are available for use by patients themselves or by health professionals [9, 10]. Obtaining risk factor information directly from patients is critical in tools such as FRAX®, but self-reported health information can be subjected to bias or simply unavailable due to unknown family history [11, 12]. If fracture risk could be accurately determined using administrative data, it could offer a faster, more robust, and egalitarian alternative, which may be a feasible option for individualised case finding or a population-level screening programme.

In this study, we evaluated the possibilities of a digital detection tool which general practitioners can use to identify individuals at high risk of major osteoporotic fractures (MOF). In 2018, we presented the Fracture Risk Evaluation Model (FREM) [13], which was developed to identify individuals at high imminent risk of hip fractures or MOFs. The difference between FREM and other risk assessment tools, such as FRAX®, is that FREM estimates an individual’s fracture risk by including significant risk factors routinely collected in register data. FREM eliminates the need for manual entry of patient-provided information, thus simplifying the process and reducing errors in data collection and entry avoiding human errors, and enabling the model to serve as a data-driven decision support tool. This approach may streamline the process of identifying individuals at risk of fractures, enhancing efficiency and enabling timely intervention strategies to reduce the burden of osteoporotic fractures [14, 15].

The major advantage of FREM is that it solely relies on administrative data, although this might also pose a significant challenge due to the omission of risk factors not included within the registers. Body mass index, early menopause, smoking habits, alcohol consumption, and familial predisposition to osteoporosis are all established clinical risk factors [16, 17]. However, these factors are not available in the registers and therefore not included in FREM. While providing equity for patients irrespective of their ability to accurately recall parental hip fractures or their own fracture history, omission of these clinical risk factors could potentially affect the risk assessment estimates produced by FREM.

Therefore, the aim of this study was to investigate whether adding self-reported clinical data on known risk factors for osteoporosis to the FREM algorithm improves precision in predicting 1-year MOF risk.

## Data and methods

### Study population

The population included in the current study originated from the Risk-stratified Osteoporosis Strategy Evaluation (ROSE) study [18]. The ROSE study was a population-based trial assessing the effectiveness of an osteoporosis screening programme, in which the fracture risk assessment tool FRAX® was used to select women with moderate-to-high fracture risk for dual-energy X-ray absorptiometry (DXA) scans [18].

In brief, women aged 65–80 years inhabiting the Region of Southern Denmark on February 1, 2010, were identified using the Danish Civil Registration System (CRS) [19], and around 35,000 were randomly selected for possible inclusion in the ROSE study. The women were invited to participate in the study between February 2010 and November 2011.

The women were randomised into either a screening group or a control group, and both groups received an invitation letter containing a questionnaire. The items in the questionnaire were used to calculate a FRAX® score, and women in the screening group with a score of ≥ 15% were offered a DXA scan. About 80% (27,157) returned the questionnaire. Of these, 6252 women returned blank questionnaires or questionnaires with missing data and were therefore excluded. Furthermore, 2300 were excluded due to already being in osteoporotic treatment. Thus, the study population in the current study comprised 18,605 women with adequate data (9279 from the screening group and 9326 controls). A flowchart based on the inclusion and exclusion criteria in the ROSE study can be found in Supplemental File 1: Fig. S1 [20]. The index date was defined as the latest date the women either received the questionnaire or a reminder or filled out the questionnaire.

### Data sources

The self-administered questionnaires contained 25 validated items capturing clinical risk factors and allowing calculation of the 10-year probability of MOF using FRAX®. Returned questionnaires were inspected for missing or extreme values, and the women were contacted by telephone for clarification if one was found [18]. The study population was linked to data from Danish nationwide registers, using the unique personal identifiers assigned to all inhabitants in Denmark, making it possible to link individual-level data [21]. Date of birth, death, and emigration were obtained from the CRS [19]. Diagnostic codes (International Classification of Diseases, 10th Revision (ICD-10)) on primary and secondary hospital diagnoses, as well as surgical procedure and radiology codes, were retrieved from the National Patient Register, which includes all inpatient and outpatient hospital contacts in Denmark [22, 23].

### Clinical risk factors

Clinical risk factors, as listed by the Danish national treatment guideline, were selected to assess whether inclusion improved the prediction afforded by the FREM algorithm [16]. These risk factors were obtained from (1) the ROSE questionnaire (self-reported data) and (2) the Danish national administrative registers. The clinical risk factors obtained from the questionnaire included the following: previous fracture sustained after turning 40 years old, parental hip fractures, current smoker (yes/no), alcohol consumption ≥ 3 units daily, body mass index ≤ 19 kg/m^2^, early menopause, use of oral glucocorticoids, increased fall risk, long-term immobilisation, rheumatoid arthritis, osteogenesis imperfecta, malabsorption (previous gastrectomy), primary hyperparathyroidism, organ transplant, Cushing’s disease, myeloma, and ankylosing spondylitis. Some of the selected clinical risk factors from the Danish national treatment guideline were not included in the questionnaire, and these were instead obtained from the National Patient Register with a 15-year lookback from the index date [16]. This was the case for anorexia (ICD-10: F50 and all subcodes hereof), chronic kidney disease (ICD-10: Z492, Z940, or Z992), and mastocytosis (ICD-10: Q822). Charlson Comorbidity Index (CCI) was calculated with a 10-year lookback from the index date [24]. Age on index date was obtained from the Danish Civil Registration System, and participants were categorised into age groups of 5-year intervals (65–69, 70–74, and ≥ 75 years), to correspond with the age groups included in FREM [13].

### FREM predictors

The FREM algorithm included 38 risk factors (diagnoses) for predicting the risk of MOF. Diagnoses were defined by ICD-10 codes from the National Patient Register with a 15-year lookback from the index date (Supplementary file 1: Table S1) [13]. These included, but were not limited to, fractures of the femur, alcohol-related disorders, and epilepsy. Diagnoses were included as dichotomous variables (yes/no) in the approaches. A FREM algorithm for predicting the risk of hip fracture has also been developed, but was not included in the current study due to a low number of hip fractures in the population [13].

### Major osteoporotic fractures (MOF) definition

A previously validated definition of MOF was used, which defined MOF as a fracture within four fracture sites, either the hip, wrist, humerus, or vertebrae, using the following ICD-10 codes: S720, S721, S722 (hip), S525, DS526 (wrist), S422, S423 (humerus), and S320, T08 (and all subcodes hereof), S220, S221, S120, S121, or S122 (clinical vertebral) [25]. A fracture was defined as an incident if one of the included ICD-10 fracture codes was registered within 7 days of a relevant radiology code (for wrist, humerus, or vertebral fractures) or a surgical procedure code (for hip fractures) (Supplementary file 1: Table S2). Consecutive fractures at the same skeletal site needed to be at least 90 days apart to be categorised as independent fractures.

## Statistics

The baseline characteristics and distribution of risk factors, collected in a 15-year lookback period from the index date, were stratified by incident fracture within 1 year (main analysis) and 5 years (sub-analysis) of follow-up. These were presented as medians with quartiles for continuous variables and counts and proportions for categorical and binary variables. Significant (*p* < 0.05) clinical risk factors were identified by logistic regression. Risk factors that were present in ≤ 5 cases in the subgroup of the total population that sustained a MOF within 1 year of follow-up were excluded.

We evaluated the predicative performance of three different approaches for predicting the 1-year risk of MOF: FREM^orig^, FREM-CRF, and CRF^only^. The FREM^orig^ approach used the FREM algorithm, the FREM-CRF approach included both the FREM algorithm and clinical risk factors, and the CRF^only^ approach only included clinical risk factors. Predictive performance was assessed using logistic regression and Cox proportional hazards models. The logistic regression model was chosen as it corresponds to the methodology applied in the original FREM development and validation studies [13–15].

As a sub-analysis, the Cox proportional hazards model was repeated for 5-year risk with the limitations discussed below. Cox regression models were applied for the long-term predictions to take the more prevalent censoring, i.e. death and migration, which occurs during 5 years of follow-up into account. Proportional hazards assumptions were evaluated by Schoenfeld residuals.

For each approach, the predictive capabilities were assessed by predicting each individual’s fracture risk from the regression models and from this estimating both positive predictive value (PPV) and negative predictive value (NPV) for a predetermined cut-off. Cut-offs of 2% and 10% for the 1- and 5-year risk of MOF, respectively, were applied, in accordance with cut-offs used in our previous studies [13, 14]. Furthermore, concordance statistics (*C*-statistics) were estimated as the area under the curve (AUC) for logistic regression models and as Harrell’s *C* for Cox models.

For the estimation of PPV and NPV in logistic regression models, the confidence intervals (CIs) were estimated by the exact binomial method, and the CIs were estimated using bootstrapping with 100 repetitions. For the estimation of PPV, NPV, and AUC in the Cox models, the CIs were estimated using jack-knife robust CIs [26].

Two sensitivity analyses were conducted. The first investigate the potential influence of being included in the screening procedure in the ROSE study, which included an invitation for a DXA scan for women with moderate to high fracture risk. In this analysis, the logistic regression models and Cox proportional hazard models described above were repeated for 1-year MOF risk, using only the final control group included in the ROSE study as the study population (*N* = 9326). This group returned the questionnaire, did not receive anti-osteoporotic treatment at the time of inclusion, and had a FRAX® score calculated but did not receive a DXA scan [18]. In the second sensitivity analysis, the FREM^orig^ was applied to all women who were randomised to the control group prior to receiving an invitation to the ROSE study, regardless of whether or not they returned the questionnaire (*N* = 17,157) [18].

Analyses were conducted using Stata version 18.0 [27].

## Results

The study population consisted of 18,605 individuals, of which 9326 were included as controls in the original ROSE study. Table 1 presents those who sustained a MOF and the distribution of baseline characteristics. Within 1 year, 280 women (1.5%) in the total population sustained a MOF, and 1361 women (7.3%) sustained a MOF within 5 years (Table 1). Only 54 women (0.3%) sustained a hip fracture during the first year of follow-up (data not shown), and the FREM algorithm predicting the risk of hip fracture was therefore not included in this paper. Those who sustained a MOF were slightly older at the time of index (median age, 71.85 (68.15; 75.96) years and 71.92 (68.42; 76.30) years for MOF within 1 and 5 years, respectively), compared to the population as a whole (median age, 70.32 (67.25; 74.45) years), and there was a higher proportion of women in the ≥ 75 year age category in those groups (32.5% and 34.1% for MOF within 1 and 5 years, respectively, compared to 23.6% in the total population) (Table 1). Women who sustained a MOF did not consistently report a higher occurrence of risk factors from the ROSE questionnaire compared to the population as a whole (Table 1). The greatest differences were observed in the following risk factors (total population, MOF within 1 year, MOF within 5 years, respectively): previous fracture since turning 40 years old (10.2%, 15.7%, 16.3%), current smoker (14.3%, 18.9%, 17.3%), increased fall risk (6.9%, 13.6%, 11.2%), and long-term immobilisation (8.3%, 13.2%, 10.8%). The distribution of risk factors obtained from registers was mostly similar among the groups, though the occurrence of CCI = 1 was higher in those who sustained a MOF within 1 year (11.4%) compared to the total population (6.9%) and those who sustained a MOF within 5 years (7.7%).

**Table 1:** Baseline characteristics in the total study population and according to incident major osteoporotic fractures within 1 and 5 years of follow-up

Risk factors	Study population		
	All women	MOF within 1 yr	MOF within 5 yr
	*N* (%) *N* = 18,605 (100)	*N* (%) *N* = 208 (1.5)	*N* (%) *N* = 1361 (7.3)
Years at index, (median (Q1; Q3))	70.32 (67.25; 74.45)	71.85 (68.15; 75.96)	71.92 (68.42; 76.30)
Years at index, categorised, *N* (%)			
65–69 yr	8525 (45.8)	94 (33.6)	474 (34.8)
70–74 yr	5695 (30.6)	95 (33.9)	423 (31.1)
≥ 75 yr	4385 (23.6)	91 (32.5)	464 (34.1)
Self-reported risk factors from ROSE questionnaire, *N* (%)			
Previous fracture since turning 40 yr old	1900 (10.2)	44 (15.7)	222 (16.3)
Parental hip fracture	2483 (13.3)	34 (12.1)	184 (13.5)
Current smoker	2659 (14.3)	53 (18.9)	235 (17.3)
Alcohol ≥ 3 units daily	210 (1.1)	< 3 (0.0)	17 (1.2)
Body mass index ≤ 19 kg/m^2^	563 (3.0)	16 (5.7)	66 (4.8)
Early menopause	3858 (20.7)	65 (23.2)	295 (21.7)
Use of oral glucocorticoids^a^	452 (2.4)	9 (3.2)	31 (2.3)
Increased fall risk	1289 (6.9)	38 (13.6)	153 (11.2)
Long-term immobilisation	1544 (8.3)	37 (13.2)	147 (10.8)
Rheumatoid arthritis	977 (5.3)	19 (6.8)	84 (6.2)
Osteogenesis imperfecta	26 (0.1)	0 (0.0)	5 (0.4)
Malabsorption (previous gastrectomy)	156 (0.8)	5 (1.8)	20 (1.5)
Primary hyperparathyroid disease	1274 (6.8)	18 (6.4)	104 (7.6)
Organ transplanted	< 3 (0.0)	0 (0.0)	0 (0.0)
Cushing’s disease	15 (0.1)	0 (0.0)	< 3 (0.0)
Myeloma	12 (0.1)	0 (0.0)	< 3 (0.0)
Ankylosing spondylitis	6 (0.0)	0 (0.0)	< 3 (0.0)
Risk factors obtained from register data, *N* (%)			
Anorexia nervosa	< 3 (0.0)	0 (0.0)	0 (0.0)
Chronic kidney disease	19 (0.1)	< 3 (0.0)	< 3 (0.0)
Mastocytosis	3 (0.0)	0 (0.0)	0 (0.0)
Charlson Comorbidity Index			
0	14,996 (80.6)	217 (77.5)	1081 (79.4)
1	1277 (6.9)	32 (11.4)	105 (7.7)
≥ 2	2332 (12.5)	31 (11.1)	175 (12.9)

*MOF* major osteoporotic fracture, *yr* year(s)

^a^Defined as a dosage of ≥ 5 mg for longer than 3 months

In logistic regression analysis, the following clinical risk factors were found to be statistically significant in predicting 1-year MOF risk: being in the age categories 70–74 or ≥ 75 compared to 65–69, previous fracture after turning 40 years old, current smoker, body mass index ≤ 19 kg/m^2^, increased fall risk, or CCI = 1 (Table 2).

**Table 2:** Logistic regression (odds ratios with 95% confidence intervals) for the 1-year risk of major osteoporotic fracture

Risk factors	Risk of MOF within one year	
	Adjusted^a^ OR (95% CI)	*p*-value
Age at index, categorised, *N* (%)		
65–69 yr	(Ref)	
70–74 yr	1.50 (1.12, 2.00)*	0.010*
≥ 75 yr	1.82 (1.35, 2.44)*	0.000*
Previous fracture since turning 40 yr old	1.45 (1.04, 2.02)*	0.030*
Parental hip fracture	0.90 (0.62, 1.29)	0.550
Current smoker	1.36 (1.00, 1.85)*	0.050*
Body mass index ≤ 19 kg/m^2^	1.72 (1.02, 2.90)*	0.040*
Early menopause	1.05 (0.79, 1.40)	0.720
Use of oral glucocorticoids^b^	1.02 (0.51, 2.04)	0.950
Increased fall risk	1.88 (1.31, 2.69)*	< 0.001*
Long-term immobilisation	1.28 (0.89, 1.85)	0.190
Rheumatoid arthritis	1.09 (0.67, 1.76)	0.740
Primary hyperparathyroid disease	0.88 (0.54, 1.43)	0.610
Charlson Comorbidity Index		
0	(Ref)	
1	1.50 (1.01, 2.24)*	0.040*
≥ 2	0.82 (0.56, 1.20)	0.300

Regression only includes clinical risk factors as explanatory variables. Risk factors which were present in ≤ 5 cases in the subgroup of the total population which sustained a major osteoporotic fracture within 1 year of follow-up were not included in the model

*MOF* major osteoporotic fracture, *OR* odds ratio, *CI* confidence interval

*Statistically significant in predicting MOF risk within 1 year (*p* < 0.05)

^a^Adjusted for age at index date

^b^Defined as a dosage of ≥ 5 mg for longer than 3 months

There was no substantial difference observed between the predictive capabilities of the FREM^orig^ and FREM-CRF approaches in terms of 1-year MOF risk (Table 3). Using logistic regression models, the FREM^orig^ resulted in an AUC of 0.63 (95% CI 0.60, 0.67), and the FREM-CRF produced an AUC of 0.64 (CI 0.61, 0.67). The results of the Cox proportional hazards analysis, which was done to provide a basis for comparing with 5-year outcomes where a logistic regression model would have been inappropriate due to cohort attrition, showed similar results (Table 3). The Cox model sub-analysis for 5-year follow-up also showed no meaningful difference between the FREM^orig^ (AUC = 0.60 (CI 0.58, 0.62)) and FREM-CRF (AUC = 0.61 (CI 0.60, 0.63)) (Table 3).

**Table 3:** Performance and predictive capabilities of regressions using the FREM^orig^, CRF^only^, and FREM-CRF in predicting risk of a major osteoporotic fracture within 1 year of follow-up

Total (*N* = 18,605)		MOF 1 yr risk (main analysis)		
Classification cut-off: 2%		FREM^orig^	CRF^only^	FREM-CRF
Logistic regression (OR (95% CI))	AUC (*C*-statistic, 95% CI)	0.63 (0.60, 0.67)	0.62 (0.58, 0.65)	0.64 (0.61, 0.67)
	PPV (95% CI)	3.10 (2.48, 3.83)	2.64 (2.10, 3.27)	3.10 (2.52, 3.76)
	NPV (95% CI)	98.76 (98.58, 98.93)	98.72 (98.53, 98.89)	98.82 (98.63, 98.98)
Cox proportional hazards (HR (95% CI))	AUC (*C*-statistic, 95% CI)	0.62 (0.58, 0.66)	0.61 (0.57, 0.65)	0.63 (0.60, 0.67)
	PPV (95% CI)	3.05 (2.44, 3.77)	2.62 (2.08, 3.25)	3.07 (2.50, 3.73)
	NPV (95% CI)	98.76 (98.58, 98.93)	98.71 (98.52, 98.88)	98.82 (98.63, 98.98)
Total (*N* = 18,605)		MOF 5 yr risk (sub-analysis)		
Classification cut-off: 10%		FREM^orig^	CRF^only^	FREM-CRF
Cox proportional hazards (HR (95% CI))	AUC (*C*-statistic, 95% CI)	0.60 (0.58, 0.62)	0.60 (0.58, 0.62)	0.61 (0.60, 0.63)
	PPV (95% CI)	13.16 (11.89, 14.50)	11.97 (10.87, 13.15)	12.60 (11.47, 13.81)
	NPV (95% CI)	93.65 (93.26, 94.03)	93.65 (93.25, 94.03)	93.78 (93.38, 94.15)

All models were adjusted for age at index

*MOF* major osteoporotic fracture, *yr* year(s), *FREM*^*orig*^ approach using only the FREM algorithm, *CRF*^*only*^ approach using only clinical risk factors, *FREM-CRF* approach combining the FREM algorithm and clinical risk factors, *CI* confidence interval

**Fig. 1:**
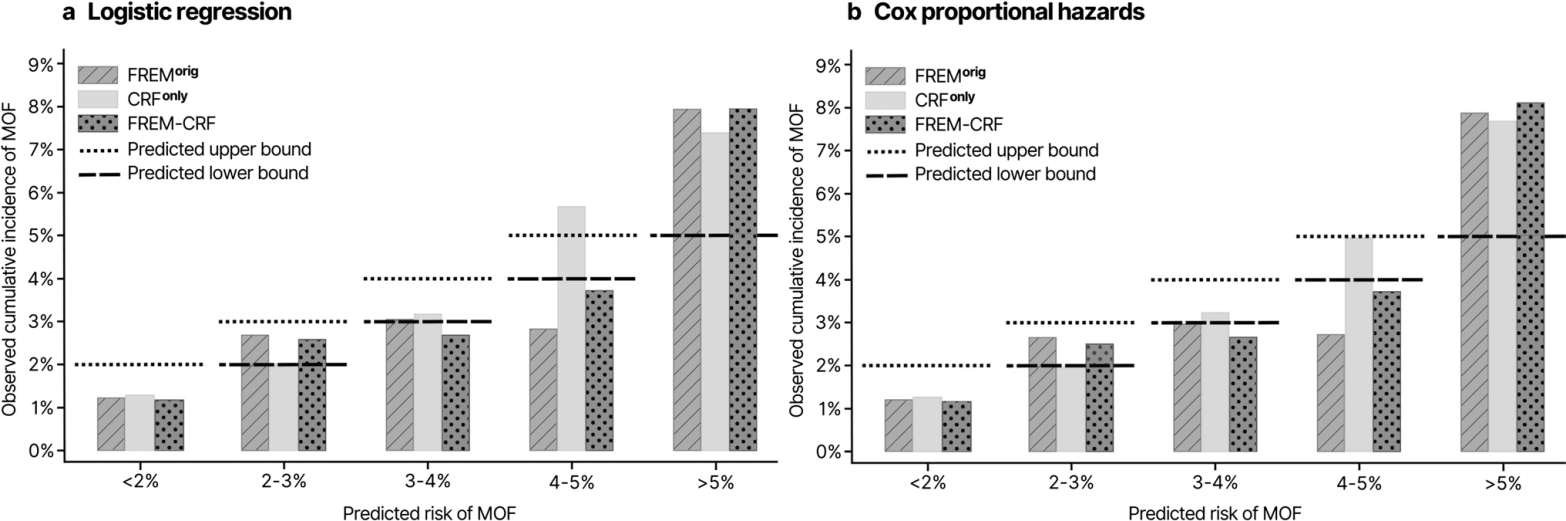
Predicted risk versus observed cumulative incidence of major osteoporotic fractures (MOF) within 1 year using logistic regression (**1a**) and Cox proportional hazards (**1b**) for FREM^orig^ (striped), CRF^only^ (no pattern), and FREM-CRF (dotted). Predicted upper bound marked by dotted line and predicted lower bound marked by striped line

The FREM^orig^ and FREM-CRF approaches were also nearly identical in their prediction of MOF within 1 year using both logistic and Cox regression analyses (Fig. 1a, b). The largest discrepancy between the models was observed at a 4–5% predicted risk of MOF, where both models underestimated the risk, whereas the CRF^only^ approach overestimated the risk.

Two sensitivity analyses were performed. The first investigated the influence of being included in the screening procedure in the ROSE study, by repeating the main analyses for the final ROSE control group. This control group displayed a similar age and risk factor distribution (both self-reported and obtained from registers) compared to the total population (Supplementary file 1: Table S3), and the analyses yielded results that were consistent with the main analysis (Supplementary file 1: Table S4). In the second sensitivity analysis, the FREM algorithm was applied to all women randomised to the ROSE control group, regardless of whether they returned the questionnaire. This analysis yielded slighter higher estimates than the main analyses across AUC, PPV, and NPV in both logistic regression and Cox models (Supplementary file 1: Table S5).

## Discussion

In this study, we evaluated the discrimination of three approaches for fracture risk prediction: one using the original FREM algorithm, one based solely on clinical risk factors, and one combining both FREM and clinical risk factors. We applied the approaches to a cohort from a Danish population-based randomised controlled screening trial.

Overall, the three approaches performed very similarly. They displayed similar AUCs/*C*-statistics, PPVs, and NPVs, with the main difference observed in the PPV, which was slightly lower for the CRF^only^ approach compared to the FREM^orig^ and FREM-CRF across both the main and sub-analysis (Table 3). In terms of predicted versus observed MOFs, the three approaches only differed markedly from each other in the 4–5% risk group, where the FREM^orig^ and the FREM-CRF underestimated the risk of MOF, while the CRF^only^ overestimated the risk. These findings suggest that administrative data can be just as effective as clinical risk factors in identifying women at high risk of MOFs when used in prediction models. The *C*-statistics for the FREM^orig^ approach were lower in this study (AUC (*C*-statistic) = 0.63 (0.60, 0.67) for MOF) compared to the original FREM study, where an AUC of 0.75 (95% CI, 0.74; 0.79) for MOF in women was observed [13]. This can likely be attributed to the narrower age span of the ROSE population and a less socioeconomically diverse group providing risk factor information. We have previously reported that non-completion of the ROSE questionnaire was associated with older age, living alone, lower education, lower income, and higher comorbidity [28].

Several studies have developed fracture risk scores using administrative data [29–31]. A German study used insurance claims data to develop a fracture risk assessment model for predicting MOF within 2 years [29]. The full model, which included age, sex, prior fracture, and some medications, achieved a *C*-statistic of 0.70 (95% CI 0.69, 0.71), and the authors reported the inclusion of other risk factors marginally improved discrimination [29]. Another study examined the discrimination of fracture risk prediction models developed using administrative data from a Canadian chronic disease surveillance database [30]. The models assessing 5-year risk of fracture achieved *C*-statistics of 0.71 (95% CI 0.70, 0.71) and 0.76 (95% CI 0.76, 0.77) for MOF and hip fractures, respectively, and performed slightly better when compared to simpler models [30]. Another Canadian study compared FRAX® with and without bone mineral density (BMD) to modified administrative FRAX® models (FRAX-A and FRAX-A^+^) [31]. Both of the administrative models achieved comparable or slightly better discrimination than FRAX® without BMD when assessing the risk of MOF [31]. While the results of these studies are not directly comparable to ours due to methodological differences, they share the commonality that they demonstrate how administrative data has the potential to be used in fracture risk prediction, with similar or even slightly improved discriminative ability compared to models based on clinical risk factors [29–31].

As the burden of osteoporotic fractures is expected to grow, it seems a necessary step to develop more efficient strategies for fracture prevention. Fracture risk assessment tools based on clinical risk factors are intrinsically limited by the human factor, as it takes time to collect and enter clinical data. It may be more efficient to utilise routinely collected administrative data, which is an important consideration as digitisation progresses [10, 32]. Risk assessment models based on administrative data lend themselves well to automation which could make them ideal for large-scale screening, but population-based screening programmes for fracture risk have long been a topic of debate [33, 34]. A systematic review combined findings from the SCOOP, SOS, and ROSE trials and showed that population-based screening could be effective in reducing fracture incidence [35]. A recent 10-year follow-up of the ROSE trial showed a 14% reduction in moderate to high-risk women who underwent a DXA scan, though there was no overall reduction in osteoporotic fractures or mortality when considering the whole population [36]. As more evidence of their efficacy and feasibility emerges, it seems a logical step to incorporate digital, automated risk prediction models into screening programmes. Additionally, this approach does not rely on the ability of patients to provide accurate information on personal risk factors, including family history, which contrasts with recall-based tools such as FRAX® where the inclusion of parental hip fracture substantially alters the recommendations according to NOGG guidelines [37]. The ability to accurately report parental fracture history may or may not come with socioeconomic bias, but taking this issue out of the equation offers a potentially more widely applicable tool. However, limitations also apply to administrative healthcare data. FREM would be less useful in fracture risk assessment for persons with little contact with the hospital system [23]. In this respect, using data from prescriptions and general practitioners might offer better risk predictions.

There are factors which could have affected our findings. Non-participation among women in the ROSE trial was 39% (13,324), and as discussed above, being a non-responder was associated with older age, living alone, lower education, lower income, and higher comorbidity [28]. In the current study, several established clinical risk factors were not associated with 1-year risk of MOF, including parental hip fracture, early menopause, and use of oral glucocorticoids. This may be due to the difference in sociodemographic characteristics between responders and non-responders, as those with lower socioeconomic status (SES) may have fewer resources and display lower health literacy scores [38]. Lower SES has been linked to a lack of knowledge about osteoporosis and less likelihood of being assessed for the condition [39]. The women included in our study might have been more likely to engage in help-seeking behaviours, such as being assessed for osteoporosis if they have a parental history of hip fracture, than their non-respondent counterparts.

### Strengths and limitations

A strength of this study is that the population originates from a large population-based randomised controlled trial with prospectively collected data. Another strength is that the ROSE trial used a validated questionnaire and that we enriched this data with data from the national health registers [18]. Register data originated from the National Patient Register, which provides comprehensive data on a range of diagnoses, including fractures [22, 25]. The ROSE study used a validated questionnaire, although the version used in the study had been modified to include four additional questions [18, 40]. We do not expect this modification to have a significant impact on validity.

A limitation of this study is that just 54% (18,605/34,229) of the ROSE population returned the questionnaire and provided sufficient clinical data to be included. As mentioned above, non-completion of the self-reported questionnaire was associated with several socioeconomic factors, and non-responders did likely represent a higher-risk population than those who returned the questionnaire [28]. Thus, our study population is not representative of the general population. Additionally, as the population only included women, the results of this study cannot be applied to men. Another limitation is the risk of recall bias as clinical data was based on self-reports, but we do not expect differential recall bias to directly influence the results as the data was collected prospectively and independently of knowledge about the outcome. Recall issues may have affected responses to the self-reported questionnaire, but these issues are likely always present in self-reported health data, including when recall-based tools such as FRAX® are used. ROSE participants were also represented in the original development of the FREM algorithm, but only represent a small contribution; still, the sample cannot be considered a true external validation cohort. Lastly, FREM was developed using data on the Danish population only. This population is fairly homogenous, and only 15.9% of the Danish population is comprised of immigrants and their descendants, including those from neighbouring countries [41, 42]. The validity of FREM in people of non-European ancestry has not been assessed. Furthermore, FREM is based on administrative data recorded in the Danish registers, which other countries may not have available in the same format, though it would be possible to adapt FREM for use in different settings.

## Conclusion

In conclusion, adding self-reported clinical data to the fracture algorithm FREM did not increase precision in identifying individuals at high risk of osteoporotic fractures, since no large differences between the FREM^orig^ and FREM-CRF were found in 1-year fracture risk prediction. Additionally, there were no large differences between the discriminative capabilities of the FREM^orig^ or FREM-CRF approaches compared to the CRF^only^, which was based solely on clinical risk factors. In other words, the FREM algorithm was able to provide identification of persons at high risk of osteoporotic fractures by using a register-based approach that was equally effective to approaches based on clinical risk factors, without relying on the ability of patients to provide accurate information on personal risk factor profile. Thus, administrative data may play an important role in future fracture prevention strategies and population-based screening programmes. Further studies are needed to validate these findings both in the general Danish population and in external populations.

## Supplementary Information

Below is the link to the electronic supplementary material.

Supplementary file1 (DOCX 151 KB)

## Data Availability

Data is not publicly available as it contains personal identifiers, and restrictions apply to the availability and use in research. Register data is available from the Danish Health Data Authority. Analaysis codes and data collected in connection to the ROSE study may be available upon request to the corresponding author KHR.

## References

[1] Kanis JA, Norton N, Harvey NC, Jacobson T, Johansson H, Lorentzon M, McCloskey EV, Willers C, Borgström F (2021) SCOPE 2021: a new scorecard for osteoporosis in Europe. Arch Osteoporos 16:82 34080059 10.1007/s11657-020-00871-9 PMC8172408

[2] Dyer SM, Crotty M, Fairhall N, Magaziner J, Beaupre LA, Cameron ID, Sherrington C (2016) A critical review of the long-term disability outcomes following hip fracture. BMC Geriatr 16:158 27590604 10.1186/s12877-016-0332-0 PMC5010762

[3] Johnell O, Kanis JA (2006) An estimate of the worldwide prevalence and disability associated with osteoporotic fractures. Osteoporos Int 17:1726–1733 16983459 10.1007/s00198-006-0172-4

[4] Christensen ER, Clausen A, Petersen TG, Skjødt MK, Abrahamsen B, Möller S, Rubin KH (2023) Excess mortality following a first and subsequent osteoporotic fracture: a Danish nationwide register-based cohort study on the mediating effects of comorbidities. RMD Open 9:e003524 38030232 10.1136/rmdopen-2023-003524 PMC10689412

[5] Kanis JA, Cooper C, Rizzoli R, Reginster JY (2019) European guidance for the diagnosis and management of osteoporosis in postmenopausal women. Osteoporos Int 30:3–44 30324412 10.1007/s00198-018-4704-5 PMC7026233

[6] Rubin KH, Abrahamsen B, Hermann AP, Bech M, Gram J, Brixen K (2011) Prevalence of risk factors for fractures and use of DXA scanning in Danish women A regional population-based study. Osteoporos Int 22:1401–1409 20683710 10.1007/s00198-010-1348-5

[7] Harvey NC, McCloskey EV, Mitchell PJ, Dawson-Hughes B, Pierroz DD, Reginster JY, Rizzoli R, Cooper C, Kanis JA (2017) Mind the (treatment) gap: a global perspective on current and future strategies for prevention of fragility fractures. Osteoporos Int 28:1507–1529 28175979 10.1007/s00198-016-3894-y PMC5392413

[8] Wells GA, Cranney A, Peterson J, Boucher M, Shea B, Robinson V, Coyle D, Tugwell P (2008) Alendronate for the primary and secondary prevention of osteoporotic fractures in postmenopausal women. Cochrane Database Syst Rev 23(1):Cd001155. 10.1002/14651858.CD001155.pub2 10.1002/14651858.CD001155.pub2 18253985

[9] Rubin KH, Friis-Holmberg T, Hermann AP, Abrahamsen B, Brixen K (2013) Risk assessment tools to identify women with increased risk of osteoporotic fracture: complexity or simplicity? A systematic review. J Bone Miner Res 28:1701–1717 23592255 10.1002/jbmr.1956

[10] Gupta A, Maslen C, Vindlacheruvu M et al (2022) Digital health interventions for osteoporosis and post-fragility fracture care. Ther Adv Musculoskelet Dis 14:1759720x221083523 35368375 10.1177/1759720X221083523 PMC8966117

[11] Spitzer S, Weber D (2019) Reporting biases in self-assessed physical and cognitive health status of older Europeans. PLoS One 14:e0223526 31593576 10.1371/journal.pone.0223526 PMC6783110

[12] Baleanu F, Moreau M, Kinnard V, Iconaru L, Karmali R, Rozenberg S, Rubinstein M, Paesmans M, Bergmann P, Body JJ (2020) Underevaluation of fractures by self-report: an analysis from the FRISBEE cohort. Arch Osteoporos 15:61 32323006 10.1007/s11657-020-00739-y

[13] Rubin KH, Möller S, Holmberg T, Bliddal M, Søndergaard J, Abrahamsen B (2018) A new fracture risk assessment tool (FREM) based on public health registries. J Bone Miner Res 33:1967–1979 29924428 10.1002/jbmr.3528

[14] Skjødt MK, Möller S, Hyldig N, Clausen A, Bliddal M, Søndergaard J, Abrahamsen B, Rubin KH (2021) Validation of the fracture risk evaluation model (FREM) in predicting major osteoporotic fractures and hip fractures using administrative health data. Bone 147:115934 33757901 10.1016/j.bone.2021.115934

[15] Möller S, Skjødt MK, Yan L et al (2022) Prediction of imminent fracture risk in Canadian women and men aged 45 years or older: external validation of the Fracture Risk Evaluation Model (FREM). Osteoporos Int 33:57–66 34596704 10.1007/s00198-021-06165-1

[16] Dansk Knoglemedicinsk Selskab Vejledning til udredning og behandling af Osteoporose. https://www.danskknogleselskab.dk/behandlingsvejledning-nbv/osteoporose/

[17] Pouresmaeili F, Kamalidehghan B, Kamarehei M, Goh YM (2018) A comprehensive overview on osteoporosis and its risk factors. Ther Clin Risk Manag 14:2029–2049 30464484 10.2147/TCRM.S138000 PMC6225907

[18] Rubin KH, Holmberg T, Rothmann MJ et al (2015) The risk-stratified osteoporosis strategy evaluation study (ROSE): a randomized prospective population-based study. Design and baseline characteristics. Calcif Tissue Int 96:167–179 25578146 10.1007/s00223-014-9950-8

[19] Pedersen CB (2011) The Danish civil registration system. Scand J Public Health 39:22–25 21775345 10.1177/1403494810387965

[20] Rubin KH, Rothmann MJ, Holmberg T et al (2018) Effectiveness of a two-step population-based osteoporosis screening program using FRAX: the randomized Risk-stratified Osteoporosis Strategy Evaluation (ROSE) study. Osteoporos Int 29:567–578 29218381 10.1007/s00198-017-4326-3

[21] Schmidt M, Schmidt SAJ, Adelborg K, Sundbøll J, Laugesen K, Ehrenstein V, Sørensen HT (2019) The Danish health care system and epidemiological research: from health care contacts to database records. Clin Epidemiol 11:563–591 31372058 10.2147/CLEP.S179083 PMC6634267

[22] Schmidt M, Schmidt SA, Sandegaard JL, Ehrenstein V, Pedersen L, Sørensen HT (2015) The Danish National Patient Registry: a review of content, data quality, and research potential. Clin Epidemiol 7:449–490 26604824 10.2147/CLEP.S91125 PMC4655913

[23] Lynge E, Sandegaard JL, Rebolj M (2011) The Danish National Patient Register. Scand J Public Health 39:30–33 21775347 10.1177/1403494811401482

[24] Quan H, Sundararajan V, Halfon P, Fong A, Burnand B, Luthi JC, Saunders LD, Beck CA, Feasby TE, Ghali WA (2005) Coding algorithms for defining comorbidities in ICD-9-CM and ICD-10 administrative data. Med Care 43:1130–1139 16224307 10.1097/01.mlr.0000182534.19832.83

[25] Clausen A, Möller S, Skjødt MK, Lynggaard RB, Vinholt PJ, Lindberg-Larsen M, Søndergaard J, Abrahamsen B, Rubin KH (2024) Validity of major osteoporotic fracture diagnoses in the Danish National Patient Registry. Clin Epidemiol 16:257–266 38633218 10.2147/CLEP.S444447 PMC11022871

[26] Newson RB (2010) Comparing the predictive powers of survival models using Harrell’s C or Somers’ D. The Stata J: Promoting communications on statistics and Stata 10:339–358

[27] StataCorp (2023) Stata statistical software: release 18 College Station, TX: StataCorp LLC. https://www.stata.com/support/faqs/resources/citing-software-documentation-faqs/

[28] Rothmann MJ, Möller S, Holmberg T et al (2017) Non-participation in systematic screening for osteoporosis-the ROSE trial. Osteoporos Int 28:3389–3399 28875257 10.1007/s00198-017-4205-y

[29] Reber KC, König HH, Becker C, Rapp K, Büchele G, Mächler S, Lindlbauer I (2018) Development of a risk assessment tool for osteoporotic fracture prevention: a claims data approach. Bone 110:170–176 29421456 10.1016/j.bone.2018.02.002

[30] Beaudoin C, Jean S, Moore L, Gamache P, Bessette L, Ste-Marie LG, Brown JP (2021) Prediction of osteoporotic fractures in elderly individuals: a derivation and internal validation study using healthcare administrative data. J Bone Miner Res 36:2329–2342 34490952 10.1002/jbmr.4438

[31] Yang S, Leslie WD, Morin SN, Lix LM (2019) Administrative healthcare data applied to fracture risk assessment. Osteoporos Int 30:565–571 30554259 10.1007/s00198-018-4780-6

[32] Javaid MK, Kanis JA, Kaufman JM, Lamy O, Matijevic R, Perez AD, Radermecker RP, Rosa MM, Thomas T, Thomasius F, Vlaskovska M, Rizzoli R, Cooper C (2022) Management of patients at very high risk of osteoporotic fractures through sequential treatments. Aging Clin Exp Res 34(4):695–714. 10.1007/s40520-022-02100-4 35332506 10.1007/s40520-022-02100-4 PMC9076733

[33] Chotiyarnwong P, McCloskey EV, Harvey NC et al (2022) Is it time to consider population screening for fracture risk in postmenopausal women? A position paper from the International Osteoporosis Foundation Epidemiology/Quality of Life Working Group. Arch Osteoporos 17:87 35763133 10.1007/s11657-022-01117-6 PMC9239944

[34] Solutions for Public Health (2019) Screening for osteoporosis in postmenopausal women. External review against programme appraisal criteria for the UK National Screening Committee. https://view-health-screening-recommendations.service.gov.uk/osteoporosis/

[35] Merlijn T, Swart KMA, van der Horst HE, Netelenbos JC, Elders PJM (2020) Fracture prevention by screening for high fracture risk: a systematic review and meta-analysis. Osteoporos Int 31:251–257 31838551 10.1007/s00198-019-05226-w PMC7010619

[36] Petersen TG, Abrahamsen B, Høiberg M et al (2024) Ten-year follow-up of fracture risk in a systematic population-based screening program: the risk-stratified osteoporosis strategy evaluation (ROSE) randomised trial. EClinicalMedicine 71:102584 38638398 10.1016/j.eclinm.2024.102584 PMC11024575

[37] National Osteoporosis Guideline Group (2021) Clinical guideline for the prevention and treatment of osteoporosis. https://www.nogg.org.uk/full-guideline. Accessed July 15th 2024

[38] Beauchamp A, Buchbinder R, Dodson S, Batterham RW, Elsworth GR, McPhee C, Sparkes L, Hawkins M, Osborne RH (2015) Distribution of health literacy strengths and weaknesses across socio-demographic groups: a cross-sectional survey using the Health Literacy Questionnaire (HLQ). BMC Public Health 15:678 26194350 10.1186/s12889-015-2056-z PMC4508810

[39] Roh YH, Lee ES, Ahn J, Kim HS, Gong HS, Baek KH, Chung HY (2019) Factors affecting willingness to get assessed and treated for osteoporosis. Osteoporos Int 30:1395–1401 30944954 10.1007/s00198-019-04952-5

[40] Rubin KH, Abrahamsen B, Hermann AP, Bech M, Gram J, Brixen K (2011) Fracture risk assessed by fracture risk assessment tool (FRAX) compared with fracture risk derived from population fracture rates. Scand J Public Health 39:312–318 21429990 10.1177/1403494811402412

[41] Statistics Denmark (2024) Immigrants and their descendants [Internet]. Statistics Denmark. https://www.dst.dk/en/Statistik/emner/borgere/befolkning/indvandrere-og-efterkommere# Accessed June 25th 2024

[42] Athanasiadis G, Cheng JY, Vilhjálmsson BJ et al (2016) Nationwide genomic study in Denmark reveals remarkable population homogeneity. Genetics 204:711–722 27535931 10.1534/genetics.116.189241 PMC5068857

