# Supplementary material for: Prediction of imminent osteoporotic fracture risk in Danish postmenopausal women—can the addition of self-reported clinical risk factors improve the prediction of the register-based FREM algorithm?
Source: Arch Osteoporos. 2025 Feb 7;20(1):21. doi: 10.1007/s11657-024-01493-1 (PMC11805794; doi:10.1007/s11657-024-01493-1)
Supplement: Supplementary file 1 — Supplementary file1 (DOCX 151 KB) [file 11657_2024_1493_MOESM1_ESM.docx]

**Supplementary file 1 for the paper “Prediction of imminent osteoporotic fracture risk in Danish postmenopausal women – can addition of self-reported clinical risk factors improve the prediction of the register-based FREM algorithm?”**

**Authors**

Emilie Rosenfeldt Christensen^a^, Kasper Westphal Leth ^a^, Frederik Lykke Petersen^b^, Tanja Gram Petersen ^a, b^, Sören Möller^a^, Bo Abrahamsen^a, c^, Katrine Hass Rubin^a, b^

a: Research Unit OPEN, Department of Clinical Research, University of Southern Denmark

b: OPEN - Open Patient data Explorative Network, Odense University Hospital, Odense, Denmark.

c: Department of Medicine, Holbæk Hospital, Holbæk, Denmark

**Corresponding author:**

Katrine Hass Rubin^a, b^

E-mail address: katrine.rubin@rsyd.dk

**Submitted to Archives of Osteoporosis**

**Table of contents**

[Figure S1: Flowchart describing the inclusion and exclusion process in the ROSE study 2](#_Toc185424972)

[Table S1: ICD-10 codes for risk factors included in FREM for predicting the risk of major osteoporotic fractures 2](#_Toc185424973)

[Table S2: Included surgical procedure and radiology codes by fracture type 2](#_Toc185424974)

[Table S3: Baseline characteristics in the total ROSE control population and according to incident major osteoporotic fractures within one and five years of follow-up 3](#_Toc185424975)

[Table S4: Performance and predictive capabilities of regressions using the FREM^orig^, CRF^only^ and FREM-CRF in prediction of major osteoporotic fracture risk within one year of follow-up in the final ROSE control population* 4](#_Toc185424976)

[Table S5: Performance and predictive capabilities of regressions applying the FREM^orig^ in predicting risk of major osteoporotic fracture within one year of follow-up in the overall ROSE population* 5](#_Toc185424977)

## Figure S1: Flowchart describing the inclusion and exclusion process in the ROSE study


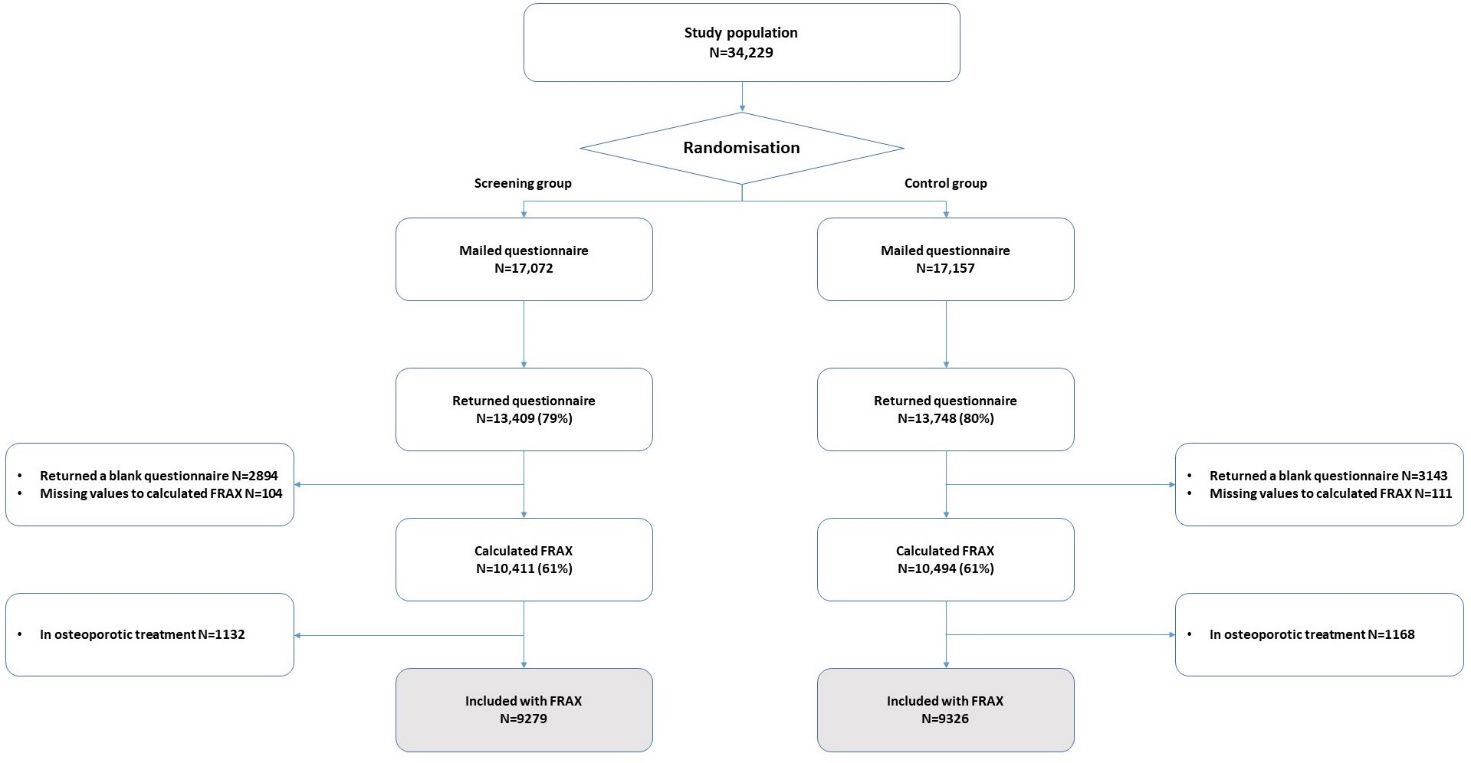


## Table S1: ICD-10 codes for risk factors included in FREM for predicting the risk of major osteoporotic fractures

| **Fracture type** | **Total ICD-10 codes** | **ICD-10 codes** |
| --- | --- | --- |
| M*OF^a^* | 38 | S52*, S42*, F10*, S72*, S22*, S92*, S80*, S32*, J44*, S62*, S82*, S01*, K70*, F00*, M81*, S93*, R41*, F20*, M06*, S63*, H36*, G40*, R63*, S30*, E86*, N62*, H50*, G54*, O80*, S90*, D51*, J13*, C77*, L89*, J90*, H47*, R67*, R26* |

a: MOF: Major Osteoporotic Fractures
* placeholder for any lower-level codes

## Table S2: Included surgical procedure and radiology codes by fracture type

| **Fracture type** | **Radiology codes (x-ray, CT and MRI scans)** | **Surgical procedure codes** |
| --- | --- | --- |
| Wrist | UXRF30, UXRF40, UXRF45, UXMF45 |  |
| Clinical vertebral | UXRE30, UXCE30, UXRE20, UXCE20, UXRE10, UXCE10 |  |
| Humerus | UXCF15, UXCF20 |  |
| Hip |  | NFB*, NFJ4*-9* |

*placeholder for any lower-level codes

## Table S3: Baseline characteristics in the total ROSE control population and according to incident major osteoporotic fractures within one and five years of follow-up

|  | ROSE control population | | |
| --- | --- | --- | --- |
|  | All women | MOF within 1 yr | MOF within 5 yr |
| Risk factors | N (%)  N=9326 (100) | N (%)  N=147 (1.6) | N (%)  N=712 (7.6) |
| Years at index, (median, (Q1;Q3)) | 70 (67;74) | 71 (68;76) | 72 (68;76) |
| Years at index, categorized, N (%) |  |  |  |
| 65-69 yr | 4319 (46.3) | 56 (38.1) | 240 (33.7) |
| 70-74 yr | 2866 (30.7) | 42 (28.6) | 227 (31.9) |
| ≥75 yr | 2141 (23.0) | 49 (33.3) | 245 (34.4) |
| Self-reported risk factors from ROSE questionnaire, N (%) | |  |  |
| Previous fracture since turning 40 years old | 919 (9.9) | 26 (17.7) | 104 (14.6) |
| Parental hip fracture | 1248 (13.4) | 20 (13.6) | 94 (13.2) |
| Current smoker | 1338 (14.3) | 27 (18.4) | 127 (17.8) |
| Alcohol ≥ 3 units daily | 111 (1.2) | <3 (0.0) | 6 (0.8) |
| Body mass index ≤19kg/m^2^ | 312 (3.3) | 11 (7.5) | 41 (5.8) |
| Early menopause | 1898 (20.4) | 31 (21.1) | 147 (20.6) |
| Use of oral glucocorticoids^a^ | 228 (2.4) | 4 (2.7) | 19 (2.7) |
| Increased fall-risk | 655 (7.0) | 19 (12.9) | 75 (10.5) |
| Long-term immobilization | 752 (8.1) | 20 (13.6) | 75 (10.5) |
| Rheumatoid arthritis | 498 (5.3) | 12 (8.2) | 42 (5.9) |
| Osteogenesis imperfecta | 12 (0.1) | 0 (0.0) | 3 (0.4) |
| Malabsorption (previous gastrectomy) | 75 (0.8) | <3 (0.0) | 9 (1.3) |
| Primary hyperparathyroid disease | 655 (7.0) | 11 (7.5) | 62 (8.7) |
| Organ transplanted | <3 (0.0) | 0 (0.0) | 0 (0.0) |
| Cushing's disease | 6 (0.1) | 0 (0.0) | 0 (0.0) |
| Myoloma | 9 (0.1) | 0 (0.0) | <3 (0.0) |
| Ankylosing spondylitis | 3 (0.0) | 0 (0.0) | 0 (0.0) |
| Risk factors obtained from register data, N (%) |  |  |  |
| Anorexia nervosa | <3 (0.0) | 0 (0.0) | 0 (0.0) |
| Chronic kidney desease | 10 (0.1) | 0 (0.0%) | 0 (0.0) |
| Mastocytosis | <3 (0.0) | 0 (0.0) | 0 (0.0) |
| Charlson Co-morbidity Index |  |  |  |
| 0 | 7450 (79.9) | 115 (78.2) | 560 (78.7) |
| 1 | 660 (7.1) | 20 (13.6) | 64 (9.0) |
| ≥2 | 1216 (13.0) | 12 (8.2) | 88 (12.4) |

MOF: Major osteoporotic fracture

yr: year(s)

a: Defined as a dosage of ≥5 mg for longer than 3 months

## Table S4: Performance and predictive capabilities of regressions using the FREM^orig^, CRF^only^ and FREM-CRF in prediction of major osteoporotic fracture risk within one year of follow-up in the final ROSE control population*

| **Controls (N=9326)** | | **MOF 1 year risk** | | |
| --- | --- | --- | --- | --- |
| Classification cut-off: 2% | | **FREM^orig^** | **CRF^only^** | **FREM-CRF** |
| **Logistic regression** | AUC  (C-statistic, 95% CI) | 0.62 (0.58,0.67) | 0.64 (0.60,0.69) | 0.66 (0.61,0.70) |
|  | PPV (95% CI) | 3.11 (2.30,4.10) | 3.08 (2.30,4.03) | 3.19 (2.44,4.09) |
|  | NPV (95% CI) | 98.73 (98.45,98.96) | 98.75 (98.47,98.99) | 98.82 (98.55,99.05) |
| **Cox regression** | AUC  (C-statistic, 95% CI) | 0.61 (0.56,0.66) | 0.64 (0.59,0.69) | 0.65 (0.60,0.70) |
|  | PPV (95% CI) | 3.10 (2.29,4.09) | 3.07 (2.29,4.02) | 3.14 (2.40,4.03) |
|  | NPV (95% CI) | 98.73 (98.45,98.96) | 98.75 (98.47,98.98) | 98.82 (98.55,99.05) |
| **Controls (N=9326)** | | **MOF 5 year risk (sub analysis)** | | |
| Classification cut-off: 10% | | **FREM^orig^** | **CRF^only^** | **FREM-CRF** |
| **Cox regression** | AUC  (C-statistic, 95% CI) | 0.61 (0.59,0.64) | 0.61 (0.59,0.64) | 0.63 (0.61,0.65) |
|  | PPV (95% CI) | 14.15 (12.49,15.95) | 11.87 (10.59,13.24) | 13.23 (11.71,14.88) |
|  | NPV (95% CI) | 93.73 (93.17,94.26) | 93.79 (93.20,94.35) | 93.72 (93.15,94.26) |

*This group returned the questionnaire and had a FRAX® score calculated but did not receive a DXA scan

All models were adjusted for age at index.

MOF: Major osteoporotic fracture

FREM^orig^: Approach using only the FREM algorithm

CRF^only^: Approahc using only clinical risk factors

FREM-CRF: Approach combining the FREM algorithm and clinical risk factors

CI: Confidence interval

## Table S5: Performance and predictive capabilities of regressions applying the FREM^orig^ in predicting risk of major osteoporotic fracture within one year of follow-up in the overall ROSE population*

| **Total (N=17,157)** | | **MOF 1 year risk** | | |
| --- | --- | --- | --- | --- |
| Classification cut-off: 2% | | **FREM^orig^** | **CRF^only^** | **FREM-CRF** |
| **Logistic regression (OR (95% CI)** | AUC  (C-statistic, 95% CI) | 0.66 (0.63,0.69) | n/a | n/a |
|  | PPV (95% CI) | 3.58 (3.08,4.14) | n/a | n/a |
|  | NPV (95% CI) | 98.75 (98.53,98.94) | n/a | n/a |
| **Cox proportional hazards**  **(HR (95% CI)** | AUC  (C-statistic, 95% CI) | 0.64 (0.61,0.68) | n/a | n/a |
|  | PPV (95% CI) | 3.59 (3.09,4.14) | n/a | n/a |
|  | NPV (95% CI) | 98.76 (98.55,98.95) | n/a | n/a |
| **Total (N=17,157)** | | **MOF 5 year risk (sub-analysis)** | | |
| Classification cut-off: 10% | | **FREM^orig^** | **CRF^only^** | **FREM-CRF** |
| **Cox proportional hazards**  **(HR (95% CI)** | AUC  (C-statistic, 95% CI) | 0.63 (0.62,0.65) | n/a | n/a |
|  | PPV (95% CI) | 14.40 (13.46,15.39) | n/a | n/a |
|  | NPV (95% CI) | 93.28 (92.82,93.73) | n/a | n/a |

*This group includes all women who were randomised to the control group, regardless of whether they returned the questionnaire

All models were adjusted for age at index.

MOF: Major osteoporotic fracture

FREM^orig^: Approach using only the FREM algorithm

CRF^only^: Approach using only clinical risk factors

FREM-CRF: Approach combining the FREM algorithm and clinical risk factors

CI: Confidence interval

n/a: not applicable
